# The development of the cognitive vestibular function scale in the elderly complaints of imbalance: a study on validity and reliability

**DOI:** 10.1016/j.bjorl.2023.101282

**Published:** 2023-06-28

**Authors:** Aysenur Ozkul, Ozlem Konukseven

**Affiliations:** a Istanbul Aydin University, Institute of Postgraduate Education, Doctorate Programme of Audiology, Istanbul, Turkey; b Istanbul Aydin University, Faculty of Health Science, Department of Audiology, Istanbul, Turkey; c Avrasya University, Vocational School of Health Services, Audiometry Programme, Trabzon, Turkey

**Keywords:** Scale, Spatial, Cognition, Cognitive, Dizziness

## Abstract

**Objectives:**

With a valid and reliable scale, it will make an important contribution to the assessment of cognitive functions such as spatial, spatial-visual, and memory functions, and raise awareness of older people with balance disorders. The purpose of this study is to develop a scale to assess vestibular and cognitive functions in the geriatric population with vestibular disorders and to evaluate its validity and reliability.

**Methods:**

The study involved 75 individuals aged 60 years and older who complained of imbalance. In the first phase, scale items on balance, emotional, spatial, spatial-visual, and memory were created using the literature. The item analysis was completed by a pilot application, and 25 scale items were determined for the main application. The item analysis and validity and reliability analyzes were completed, and the scale was given its final form. For the statistical analysis of the data, a principal component analysis was performed for the validity analysis. The Cronbach alpha coefficient was used for the reliability analysis. Descriptive statistics were compiled on the participants' scale scores.

**Results:**

The Cronbach's alpha value of the scale was found to be highly reliable at 0.86. Statistically significant values were found between the age variable and the spatial subscales, the spatial-visual subscales, and the Cognitive Vestibular Function Scale with a small positive effect (respectively: *r* = 0.264; *p* =  0.022; *r* = 0.237; *p* =  0.041; *r* = 0.231; *p* =  0.046). The results indicate that the Cognitive Vestibular Function Scale is a valid and reliable measurement tool at a good level in elderly people aged 60 years and older.

**Conclusion:**

Cognitive Vestibular Function Scale; was developed to detect cognitive problems related to dizziness/balance. As a result, a preliminary study was conducted to investigate a rapid, easy-to-use, and reliable clinical scale to assess cognitive function in people with balance disorders.

Level II Randomized trials Prospective comparative study.

## Highlights

• Clinical and laboratory studies show the relationship of vestibular inputs to various high functions.
• Cognitive Vestibular Function Scale is developed to detect cognitive problems associated with vertigo/imbalance.
• The basic component analysis is used to evaluate the validity of the scale Cronbach alpha coefficient for its reliability.

## Introductıon

Cognitive function is analyzed in more detail by dividing it into a series of cognitive domains.^1^ These domains are explained under the subtitle’s visuospatial cognition, spatial memory, navigation, and temporal memory. In addition, findings from animal and human studies conducted in recent years show that not only hearing loss, but also vestibular loss can have an impact on cognitive functions.^2^ It has been shown that when there is disharmony between the vestibular system and other sensory information, the theta rhythm in the hippocampus increases.^3^ Although the strongest link between vestibular function and the cognitive domain is in visuospatial abilities, including spatial memory, navigation, mental rotation, and mental representation of three-dimensional space, there is also important evidence that the vestibular system has an impact on attention and cognitive processing ability.^1^ The function of the vestibular organ decreases with age; older people with impaired vestibular function have been shown to perform worse on neurocognitive tests of spatial perception. Thus, vestibular losses that occur because of aging also impair spatial navigation.^4^ In reviewing the literature, scaled studies examining vestibular and cognitive functions related to dizziness and imbalance are considered insufficient.^5^ This study aims to develop a scale to assess vestibular and cognitive functions for the complaint of imbalance in the geriatric population.

## Methods

This is a randomized cross-sectional study. This study was approved by the Ethics Committee for Non-interventional Clinical Research at Istanbul Aydın University (2021/434). Consent was obtained from all participants in the study. Seventy-five people aged 60 years and older who complained of imbalance participated in the study. Participants were asked: “Do you have dizziness or balance problems?” if they said “yes”, they were included in the study. They were also asked, “Do you have any known neurological conditions?” If they said “yes”, they were not included in the study. We created the Cognitive Vestibular Function Scale (CVFS) to study the effect on the cognitive system of a disturbance in balance thought to be caused by the vestibular system. This study is a preliminary study planned in three phases. In the first phase of the study, the CVFS was developed. In the second phase, the developed 25 item scale was applied to the sample group and the validity and reliability of the scale were investigated in older people with balance disorders. Finally, a statistical analysis of the data was performed, and the results obtained were reported.

The scale CVFS was created from a total of 5 predefined subscales, namely: Vestibular (V), Spatial Memory (SM), Spatial-Visual Memory (SVM), Temporal Memory (TM), and Emotional (E) state. Prior to the scale, participants were presented with a form asking about their demographic data and general health. At the beginning of the study, publications on the vestibular and cognitive systems were reviewed in the literature, experts were consulted, patients' testimonies about the complaints were considered, and the items of the scale were created considering the daily activities of the geriatric population. By creating an item pool, the 39-item scale design was subjected to the opinions of five experts to determine content validity, and the Davis technique was applied. The items of the scale were presented to five experts for content validity. The Content Validity Index (CVI) was calculated. Accordingly, the experts rated each item using a 3-point Likert scale (1 = Required, 2 = Useful but not required, and 3 = Not required). The pilot application was conducted with the final items on a similar group of participants as the selected study participants. Reverse ratings and item repetition were used for some questions in the scale to increase participant attention. The study was then launched. The item analysis was completed by a pilot application, and 25-scale items were determined for the main application. The scale was applied in the form of reading and answering by individuals. Each item was scored between 1 and 5 using the Likert system (1 = Never; 5 = Always).

Statistical analysis is performed using SPSS, version 22 (IBM SPSS Statistics for Windows, Armonk, NY; IBM Corp., Release 2013). First, the control of the normal distribution of the data was investigated with the Kolmogorov-Smirnov and the Shapiro-Wilk test. For data that were not normally distributed in binary group comparisons, the Mann-Whitney *U* test was used from non-parametric tests. The relationship of the continuous variables to each other was examined with the Spearman correlation coefficient. For the structural validity of the scale, a principal component analysis with an explanatory factor analysis was performed. The Cronbach's alpha coefficient was used for the reliability of the scale. The descriptive statistics reported are mean, standard deviation, minimum and maximum, frequency, percentage, and *r*_s_ values. The significance value *p* <  0.05 was considered in the statistical analysis.

## Results

It was predicted that the screening scale we aimed to develop would consist of approximately 20–25 items. Since there was no power analysis method for a newly developed scale, it was decided to reach 3 times the number of items, which was a total of 75 participants.^6^ Among these 75 people who volunteered to participate in the study, 49 of them were female (62.3%), and 29 were male (38.7%). The mean age of the study group was 67 ± 8.7.

The items of the candidate scale, which comprised a total of 25-items, were presented to five experts for content validity. The CVI was calculated and ranged from 0.99‒1. Accordingly, there were 7-items in the domain of V functions, 5 in the domain of SM and E, and 4 in the domain of SVM and TM (25-items in total). The experts thought that the questions of the measurement instrument were intended to provide information about the participants studied.

For the internal consistency of the CVFS Cronbach's alpha coefficient was calculated. After the calculation, it is shown that the extraction of the emotional subscale would increase the Cronbach's alpha value from 0.678 to 0.867. Therefore, the five-item emotional scale with items such as “I feel lonely.” and “In my daily life, I worry about my imbalance/dizziness when I go out without someone by my side.” was removed. Thus, the new Cronbach's alpha coefficient calculated with 20-items proved to be very reliable (0.867 > 0.8). The calculated Cronbach's alpha coefficients are given in Table 1.

**Table 1:** Cronbach's alpha coefficients of CVF scale items.

No. of items	Cronbach's alpha	Cronbach's alpha if item deleted
I. 1 (V)	0.650	0.858
I. 2 (V)	0.635	0.855
I. 3 (V)	0.645	0.856
I. 4 (V)	0.644	0.855
I. 5 (V)	0.622	0.851
I. 6 (V)	0.646	0.857
I. 7 (V)	0.655	0.864
I. 8 (E)	0.737	–
I. 9 (E)	0.714	–
I. 10 (E)	0.716	–
I. 11 (E)	0.720	–
I. 12 (E)	0.732	–
I. 13 (SM)	0.694	0.882
I. 14 (SM)	0.649	0.857
I. 15 (SM)	0.658	0.862
I. 16 (SM)	0.668	0.866
I. 17 (SM)	0.645	0.859
I. 18 (SVM)	0.644	0.858
I. 19 (SVM)	0.656	0.861
I. 20 (SVM)	0.648	0.858
I. 21 (SVM)	0.659	0.861
I. 22 (TM)	0.653	0.860
I. 23 (TM)	0.650	0.863
I. 24 (TM)	0.680	0.875
I. 25 (TM)	0.639	0.857
Total (CVFS)	0.678	0.867

The Kaiser-Meyer-Olkin (KMO) test showed that the sample size was sufficient for factor analysis. The KMO coefficient, which was KMO = 0.74, showed that the sample size was sufficient to explain the study (KMO = 0.742 > 0.60). The relationship between the factors was 1225.884 with Bartlett's test (*p* <  0.001). The KMO and Bartlett Test of Sphericity values for the CVFS are shown in Table 2.

**Table 2:** Kaiser-Meyer-Olkin and Barlett test values of CVF scale.

Kaiser-Meyer-Olkin Sample Fit Measure		0.742
Bartlett Test of Sphericity	Approx. Chi-Square	1225.884
	df	300
	Sig.	<0.001

**Figure 1:**
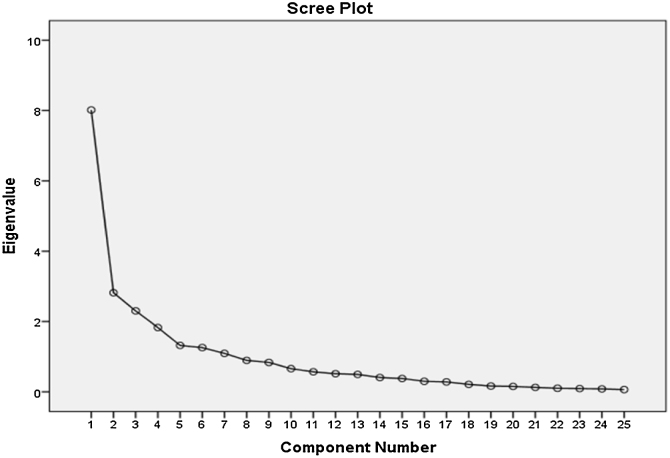
Scree Plot curve obtained by factor analysis.

Varimax rotation method was used to reduce complexity in factors by maximizing the variance of explanatory factor analysis factors. As a result of this process, it was observed that 7 factors were formed on the scale and that they explained 75.5% of the total variance. The total variance description ratio for each is given in the table. In the Scree Plot curve, the eigenvalue falls below 1 after the fifth factor. For this reason, the created scale is suitable for the formation of 5 groups. The decrease in this Scree Plot wave is shown in Fig. 1.

The substances collected because of factor analysis did not theoretically complement each other. Thus, the individual variance values of the subscales were examined by performing the principal component analysis and calculating Cronbach's alpha values of each subscale. The principal component analysis was done with the R command. The table shows the percentages of variances and Cronbach's alpha values obtained by the principal component analysis method of subscales. The results showed that two of the subscales explained more than 50% of the variance (vestibular and temporal memory). Cronbach's alpha resulted in a high degree of reliability from the vestibular subscales (0.879 > 0.8). Other subscales have defined satisfactory internal consistency. The Cronbach alpha values of the subscales and the CVFS as well as the variance values resulting from the principal component analysis are shown in Table 3.

**Table 3:** Percentage of variance explained in subscales and Cronbach's alpha values.

Scales	Percentage of variance explained (%)	Cronbach alpha	Items
Vestibular	59.696	0.879	7
Emotional	40.967	0.435	5
Spatial Memory	43.528	0.619	5
Spatial-Visual Memory	48.362	0.582	4
Temporal Memory	50.189	0.628	4
CVFS	74.556	0.879	20

Extraction Method: Principal Component Analysis.

Rotation Method: Varimax with Kaiser Normalization.

As a result of the Kolmogorov-Smirnov test, the differences were examined by comparing non-parametric tests with Mann Whitney *U* analysis because the data did not show normal distribution. The relationship between the Spearman correlation coefficient and continuous variables was examined. With increasing age, a weak linear significance was found between the UB scores, a moderately strong linear significance between the UGB scores and the total scores of the CVFS (*r* = 0.264, *p* =  0.022; *r* = 0.237, *p* =  0.041; *r* = 0.231 *p* =  0.046, respectively). For the V-scores, linearly weakly significant between the UB and TB scores, linearly moderately significant between the UGB scores, between the CVFS scores received very strong statistically significant responses (*r* = 0.399, *p* <  0.01; *r* = 0.648, *p* <  0.01; *r* = 0.281, *p* =  0.014; *r* = 0.796 *p* <  0.01, respectively). For the SM values, statistically linear, moderately significant responses were obtained between the SVM and the TM; and between CVFS obtained very strong significant responses (*r* = 0.530, *p* <  0.01; *r* = 0.427, *p* <  0.01; *r* = 0.704 *p* <  0.01, respectively). Moderately statistically significant responses were obtained between the results of TM and the CVFS (*r* = 0.683, *p* <  0.01). All the relationships between scores, and ages analyzed using the Spearman correlation coefficient are shown in Table 4.

**Table 4:** Correlation Values of Participants' Age and Scale scores.

*n* = 75		V	SM	SVM	TM	CVFS
Age	*r*_s_	0.23	0.264^a^	0.237^a^	0.114	0.231^a^
	*p*	0.05	0.022	0.041	0.329	0.046
V	*r*_s_		0.399^b^	0.648^b^	0.281^a^	0.769^b^
	*p*		<0.001	<0.001	0.014	<0.001
SM	*r*_s_			0.530^b^	0.427^b^	0.704^b^
	*p*			0.001	0.001	0.001
SVM	*r*_s_				0.629^b^	0.813^b^
	*p*				<0.001	<0.001
TB	*r*_s_					0.683^b^
	*p*					<0.001

a Correlation is significant at the 0.05 level (2-tailed).

b Correlation is significant at the 0.01 level (2-tailed).

The Mann Whitney *U* test was used to analyze all descriptive statistics, score the scales, and test for significant differences by sex. A statistically significant difference was found between the mean V-scores by gender (*z* = −2.453; *p* =  0.14). When comparing the mean V-scores of females and males, the mean V-score of males was statistically significantly lower than that of females. There was no statistically significant difference between the mean scores of the subscales SM, SVM, TM and the CVFS by gender (*p* >  0.05).

The CVFS assesses seven substances to the five substances of spatial memory, four substances of spatial-visual memory, four substances of temporal memory. Responses to the items were scored as “Always” with 5-points, “Often” with 4-points, “Sometimes” with 3-points, “Rarely” with 2-points, and “Never” with 1-point on the 5-point Likert scale. In this case, a total score of 100 points was calculated: 35 points on the vestibular subscale, 25-points on the spatial memory subscale, 20-points on the spatial-visual memory subscale, and 20-points on the temporal memory subscale. The increase in the scale score indicates that the disorder is excessive. The classification of the scores obtained from the subscales and the CVFS is shown in Table 5. The items of the CVFS are shown in Table 6.

**Table 5:** Statistical values of age and scale score averages by gender.

	Mean ± SD	Min.	Max.	*n*
Vestibular	16.35 ± 6.622	7	32	75
Spatial memory	10.87 ± 3.504	5	21	75
Spatial-visual memory	7.67 ± 2.663	5	16	75
Temporal memory	10.36 ± 3.447	4	19	75
CVFS	45.24 ± 12.845	4	86	75

**Table 6:** The cognitive vestibular function scale.

Tick your involvement in the following statements according to the scoring system alongside. Please leave nothing unmarked.					
	Always	Often	Sometimes	Rarely	Never
Vestibular					
1 I suffer from vertigo (in the form of environmental rotation).					
2 When I stand still, I feel an imbalance (trembling/swaying).					
3 When I left my head or tilt down, I feel imbalance/dizziness.					
4 When I walk, I feel like I am going left or right.					
5 I feel unbalanced/dizzy when I walk.					
6 I suffer from imbalance/dizziness in the presence of fluorescent light/bright lighting.					
7 I walk comfortably in the dark.*					
Total Score for Each Column					
Total Points					
Spatial Memory					
8 I easily estimate the distance between my location and an object/person.*					
9 When I close my eyes, I find it difficult to describe a familiar environment.					
10 When I close my eyes, I find it difficult to tell you what is new in my surroundings.					
11 I find it hard to find my car in the big car parks.					
12 When I wake up at night, I have difficulty finding the kitchen/toilet in the dark.					
Total Score for Each Column					
Total Points					
Spatial-Visual Memory					
13 I have difficulty remembering where I put certain items (controllers, phones, etc.).					
14 Magazines or newspaper pages confuse me when I read.					
15 I have difficulty recognizing familiar places (grocery shops, bakeries, etc.).					
16 I find it difficult to get to know relatives or friends.					
Total Score for Each Column					
Total Points					
Temporal Memory					
17 I find it difficult to remember my appointments, e.g., at the doctor's, at friends', etc.					
18 I forget birthdays and special occasions.					
19 I know what day of the week I am.*					
20 I am distracted/unaware during the day.					
Total Score for Each Column					
Total Points					

* Indicates reverse-scored items.

## Discussion

In this study, a scale containing twenty-five items for the vestibular and cognitive system was applied through Google Forms. “Cognitive Vestibular Function Scale” was created with the analysis of the collected data. The validity and reliability of this scale were evaluated in individuals aged 60 and over who have dizziness or imbalance complaints. When evaluating the structure validity of the CVFS, the explanatory factor analysis was used and it was observed that the Scree Plot graph had a 7-factor structure validity as 5 + 2 (5 breaking points + 2, over 1 component). However, since subscales have already been defined, the “Principal Component Analysis” method was used for the structure validity of the subscales by referencing the methodology of Lacroix et al.^7^ The individual variance values of the subscales created are examined by this method. Lacroix et al.^7^ recognized 50% as a benchmark for variances of subscales while developing the “Neuropsychological Vertigo Scale” in its study. In our study, it was observed that two of the four subscales (V and TM scales) explained more than 50% of the variance after the principal component analysis was performed. The variance values of the E, SM, and SVM subscales were lower (40% and above) than the other subscales yet still in the acceptable range.

Cronbach's alpha coefficients were examined to calculate the reliability within the scope of internal consistency of the total scale and the subscales.^7^, ^8^ Cronbach's alpha coefficient, calculated with a total of 25 items of the CVFS, was 0.678. When the Emotion subscale was removed from the scale, Cronbach's alpha coefficient increased and the new Cronbach's alpha coefficient, calculated after subtracting the five-item Emotional subscale, was found to be 0.86. Cronbach's alpha values of the Emotional subscale also showed lower internal consistency. On the other hand, the V subscale showed good internal consistency. On a TM subscale, a satisfactory internal consistency has been defined. SM and SVM subscales were found to be less consistent, although, it is acceptable for subscales with four or three items to have a lower Cronbach's alpha value.^7^ As a result of the analyzes, the validity, and reliability of the CVFS have been proven and the reliability of the scale has been obtained to a high degree. Thus, it is thought that the CVFS created by the study will contribute to the literature in terms of the evaluation of vestibular and cognitive systems together.

The review of the literature points to the work of Lacroix and his colleagues,^7^ which was produced for a similar purpose as our study. In the study, they applied the Neuropsychological Vertigo Scale, which they developed in 108 people with dizziness. As a result of the validity and reliability assessment, a scale of 28 items was formed. After the factor analysis, it is divided into 7 factors and named as time perception, position perception, memory, attention, emotional state, visual state, and motor skills. Similarly, in the developed CVFS, V, SM, SVM, and TM are separated by 4 subscales and additionally, are more specific in terms of cognitive evaluation. The scale’s subscales evaluated according to the results of Lacroix and his colleague’s study have proven to be interrelated and have shown that the vestibular and cognitive systems affect each other. After the CVFS was applied, the intensity scales of the subscales were examined according to the responses given by the participants. In this case, when the Intensity Scale was examined, it is observed that in the SVM, the normal degree range was found to be higher. After applying the CVFS, the Intensity Scales of the subscales were examined based on the participants' responses. In this case, an examination of the Intensity Scale revealed that the SVM subscale has an intensity in the normal range.

The study by Bigelow et al. included vestibular and visual-spatial evaluations. Vestibular function was measured using stimulated vestibular myogenic potentials. Visual-spatial cognitive tests include Card Rotations, Purdue Pegboard, Benton Visual Retention Test and Tracking Test Part A and B. Execution function, memory, and attention tests were also considered. As a result of the evaluations, an important relationship was found between vestibular function and visual-spatial ability and perhaps functioning memory.^9^ The vestibular function is also important for the relationship between age and cognitive decline. In the current study, unlike these results, the normal score range was obtained with intensity on a subscale that questioned Spatial Visual ability in the elderly group with imbalance complaints. Hence, additional studies are required for further examination. When the Intensity Scale was examined, intensity in the normal degree range was observed on the Spatial Memory subscale.

The study by Brandt et al. included two groups of ten subjects each, namely a patient group (four women, six men; mean age: 38) and gender and age-matched control group with no known neurological history. The bilateral vestibular nerves of all patients have been removed. Magnetic resonance imaging was evaluated using a volumeter. Participants exhibited deficiencies in performing spatial memory tasks.^10^ Parkin et al. applied temporal differentiation and frontal lobe tasks to the participants. No age effect has been observed for spatial memory. As a result, they concluded that spatial memory is affected differently from age. In the current study, the intensive obtaining of spatial memory in the normal scoring range in the spatial memory subgroup is compatible with the fact that spatial memory in the literature is not affected by age.^11^ The reason for spatial memory results is usually within the normal range in those who experience imbalance, as bilateral vestibulopathy is a more severe disorder.

Symptoms of cognitive impairment associated with vestibular disorders have long been recognized. To this end, there is a need to develop a scale to assess cognitive function in patients with complaints involving the vestibular system.^1^, ^12^, ^13^ Despite increasing literature on the role of vestibular function in visual-spatial cognitive processing, very few studies have investigated subjective cognitive complaints of vertigo patients in a single tool. Instead, most scales that investigated vertigo evaluated their physical symptoms and their impact on the patient's quality of life, mostly emotionally. DHI is the most used scale in vertigo, but it contains only a few questions about cognitive complaints. Other scales are used to assess overall patient quality of life that is not specific to vertigo or dizziness.^14^

## Conclusion

The CVFS has been developed to detect cognitive problems associated with dizziness/imbalance. In this study, a new scale capable of detecting specific cognitive dysfunction in people with balance disorders was developed, providing a rapid, easy-to-use, and reliable clinical tool. The Cronbach's alpha coefficients were examined for the reliability of the scale in the context of internal consistency. The 20-item CVFS was found to be reliable when the emotion subscale was removed, as it appeared to reduce the internal consistency of the overall scale. Consequently, the CVFS is an original scale developed to capture cognitive structures that may be affected by vestibular disorders.

## Lımıtatıons of the study

Since the number of participants is small, the CVFS should be performed on a larger sample group before clinical implementation is started. Since there are not enough articles in the field, the substances of the scale should be supported by objective tests by applying them to patients with diagnosed hearing loss and vestibular dysfunction.

## Funding

This research did not receive any specific grant from funding agencies in the public, commercial, or not-for-profit sectors.

## Conflicts of interest

The authors declare no conflicts of interest.
